# Low-concentration, continuous brachial plexus block in the management of Purple Glove Syndrome: a case report

**DOI:** 10.1186/1752-1947-4-48

**Published:** 2010-02-10

**Authors:** Georgene Singh, Verghese T Cherian, Binu P Thomas

**Affiliations:** 1 Department of Anaesthesiology, Christian Medical College, Vellore 632 004, Tamil Nadu, India; 2 Dr Paul Brand Centre for Hand Surgery, Christian Medical College, Vellore 632 004, Tamil Nadu, India

## Abstract

**Introduction:**

Purple Glove Syndrome is a devastating complication of intravenous phenytoin administration. Adequate analgesia and preservation of limb movement for physiotherapy are the two essential components of management.

**Case presentation:**

A 26-year-old Tamil woman from India developed Purple Glove Syndrome after intravenous administration of phenytoin. She was managed conservatively by limb elevation, physiotherapy and oral antibiotics. A 20G intravenous cannula was inserted into the sheath of her brachial plexus and a continuous infusion of bupivacaine at a low concentration (0.1%) with fentanyl (2 μg/ml) at a rate of 1 to 2 ml/hr was given. She had adequate analgesia with preserved motor function which helped in physiotherapy and functional recovery of the hand in a month.

**Conclusion:**

A continuous blockade of the brachial plexus with a low concentration of bupivacaine and fentanyl helps to alleviate the vasospasm and the pain while preserving the motor function for the patient to perform active movements of the finger and hand.

## Introduction

Intravenous administration of phenytoin can result in soft tissue injury at the site of injection leading to oedema and purplish-black discolouration of the hand. This is known as the Purple Glove Syndrome (PGS). The management of PGS is mainly conservative, which includes limb elevation and physiotherapy. Use of low concentration of local anaesthetic for brachial plexus block has the added advantage of preserving motor function to facilitate physiotherapy in addition to providing adequate analgesia and relief of vasospasm.

## Case presentation

**Figure 1:**
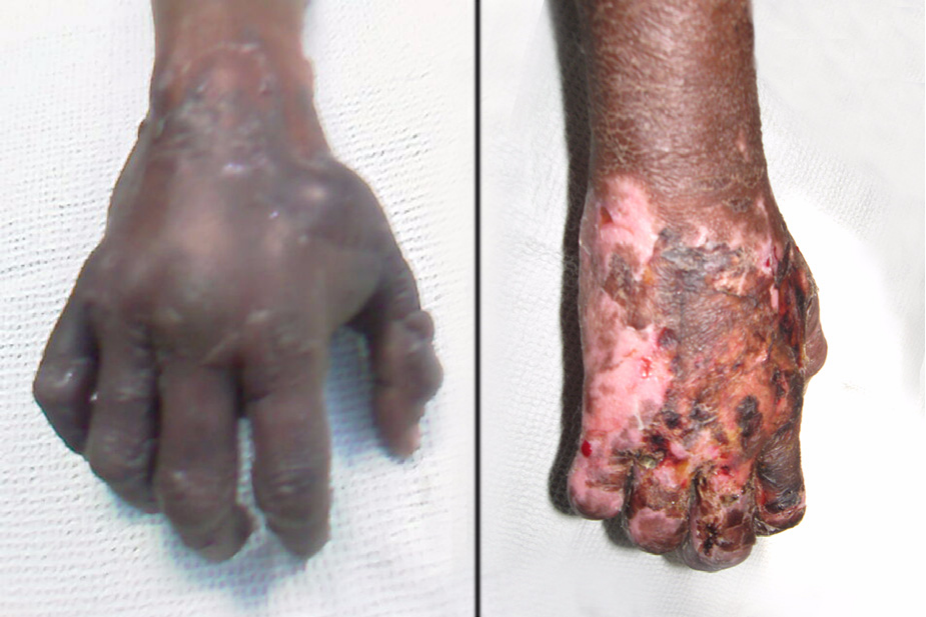
**Patient's right hand (1a) At the onset of Purple Glove Syndrome and (1b) One month later demonstrating complete recovery of flexion**.

A 26-year-old Tamil woman from India presented with an alleged history of generalized seizures. The emergency-room physician administered 600 mg of phenytoin-sodium dissolved in 500 ml of normal saline through a 20G cannula sited into a vein on the dorsum of her right hand. Four hours later, the patient complained of pain at the site of injection, which progressively became severe. The fingers, hand and forearm were swollen and had a purplish-black discolouration (Figure 1a). The radial artery was palpable, albeit feeble, under the oedema. The capillary refill under the nail bed was sluggish. The ultrasonic Doppler study of the arm showed normal flow through the radial and the ulnar arteries but the veins appeared collapsed.

The working diagnosis was that the patient had an ischemic hand, with the likelihood of progression to gangrene. This was possibly due to the extravasation of phenytoin leading to PGS. Although a differential diagnosis of compartment syndrome and need for fasciotomy to relieve the pressure was considered, it was decided to manage conservatively.

The intravenous cannula was removed and the arm was wrapped in a dry cotton-gauze dressing and kept elevated to reduce the oedema. Since the pain was intense and not relieved with non-steroidal anti-inflammatory drugs, the stellate ganglion was blocked using 7 ml of 0.5% bupivacaine. This sympathetic blockade improved the capillary refill and the mottled discolouration, and significantly reduced the pain. However, it lasted for only three hours.

**Figure 2:**
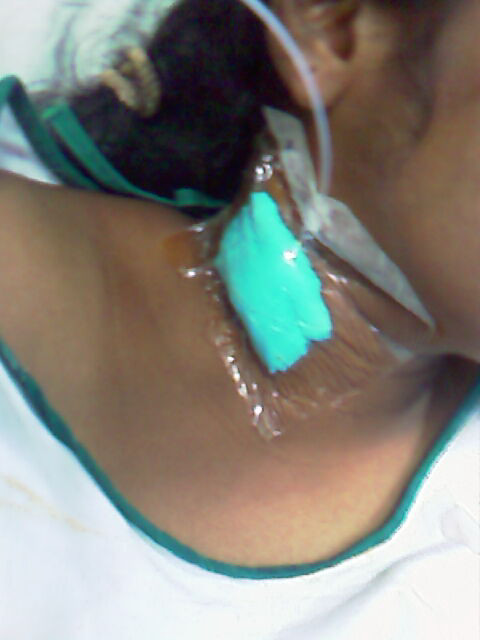
**20 G intravenous Cannula inserted into the interscalene groove for continuous infusion of bupivacaine (0.1%) with fentanyl (2 μg ml-1)**.

Therefore, it was planned to provide a continuous brachial plexus block. A 20G intravenous cannula was inserted into the interscalene grove 2.5 cm above the clavicle and was directed distally to lie within the sheath of the brachial plexus (Figure 2). A solution of 0.1% bupivacaine with fentanyl (2 μg ml^-1^) was infused at a rate of 1-2 ml.hr^-1 ^using a Terumo TE-311 infusion pump. The patient had adequate pain relief without any motor weakness. The swelling and the discolouration continued to improve with limb elevation, physiotherapy and oral antibiotics. As there was sufficient improvement, the cannula was left in-situ for seven days to provide analgesia and aid in physiotherapy. The patient was discharged from the hospital after ten days with advice to continue physiotherapy. By the end of a month, the blackish discolouration had disappeared and the range of movement of the hand was nearly back to normal (Figure 1b).

## Discussion

PGS, named for its distinctive discolouration and swelling of the hands is a known complication of intravenous administration of phenytoin-sodium [1]. It is characterized by intense pain, purplish black discolouration and oedema at the site of injection which progresses to rest of the limb [2]. The reported incidence among patients is 1-7% after intravenous injection of phenytoin [1,3,4]. It should be differentiated from extravasation of intravenous fluid, injection site infection and intra-arterial injection. The persistence of the pain, blackish discolouration and the oedema even after discontinuation of the intravenous fluid and removal of the cannula differentiates it from extravasation. The lack of purulent discharge or pyrexia differentiates it from injection site infection. Intra-arterial injection of a highly alkaline solution would lead to arterial spasm, embolisation of insoluble drug crystals, endothelial damage and vascular thrombosis resulting in the absence of Doppler signal from the artery [5].

Phenytoin is a weak acid and is insoluble in water [3]. However, injectable preparation is highly alkaline as it contains 45% propylene glycol as the solvent and 10% alcohol in water with sodium hydroxide to adjust the pH to 12 [1]. The pathophysiology of PGS is poorly understood [3]. It has been suggested that the alkaline drug precipitates upon contact with blood and leaks out of the vein, from around the cannula, and into the interstitial tissue [1]. This is likely to happen in a slow flowing stream or if the cannula is kinked, leading to stasis. Another possible mechanism could be that the highly alkaline solution induces vasoconstriction of the vein resulting in disruption of the endothelial-intercellular junctions and seepage of the drug into the interstitial space [1]. Extravasation of the highly albumin-bound (70-90%) phenytoin increases the interstitial oncotic pressure leading to oedema [1]. Propylene glycol with its high osmolality causes necrosis of the tissue [1]. Although these explanations seem plausible, reports of PGS occurring after the oral administration of phenytoin [6] also suggest that the phenomenon may be due to phenytoin itself and not directly due to the infusion [7].

Women and the elderly are said to have an increased risk of PGS. Other factors associated with it include peripheral vascular disease and diseases that weaken the vascular and dermal integrity, use of intravenous catheters smaller than 20G and infusion of phenytoin at more than 25 mg.ml^-1 ^[1,8].

Therefore, administration of phenytoin should be into a free-flowing infusion line, through a large bore intravenous catheter sited into a large vein of the forearm, in a concentration of 10 mg.ml^-1^, and at a rate not exceeding 50 mg.min^-1 ^[9]. Any evidence of venous irritation such as pain, oedema and erythema warrants immediate discontinuation of the infusion and removal of the intravenous catheter [1].

The diagnosis of PGS is based on the characteristic clinical findings and a high index of suspicion when it occurs after the administration of phenytoin. The management is mainly conservative (limb elevation, physiotherapy, control of pain, reassurance to the patient) and should be directed at minimizing the degree of soft tissue damage [1]. The affected arm should not be used for venepuncture or blood pressure measurement.

Arterioles, smaller arteries and peripheral veins are normally under vasoconstrictor influence by the alpha receptors. In addition to the vasoconstriction caused by the highly alkaline solution, pain and anxiety also cause a marked increase in arteriolar vasoconstriction mediated by the sympathetic nervous system. This results in increased resistance, reducing cutaneous perfusion. In addition, there is an associated increase in vascular tone which decreases the compliance of the venous system, reducing its blood content and increasing the venous pressure. Sympathetic blockade, by blocking the alpha receptors, improves blood flow in vasospastic disorders [10]. Stellate ganglion blockade has the advantage of blocking the sympathetic innervation of the upper limb, thus improving the perfusion and relieving the ischaemic pain associated with vasospasm [11].

Because of tissue injury and ischaemia, PGS is very painful. A low concentration local anaesthetic would relieve the pain by preferentially blocking the Aδ and B fibres [12]. Impulses in small fibres are blocked faster than those in larger ones because of the amount of time of drug diffusion and the length of the nerve to block propagation of nerve impulses. In separate experiments, it has been shown that nerve signals associated with both beta fibres and A delta fibres are reduced at low concentration of local anaesthetics [13]. Electrophysiologic studies have shown that bupivacaine diffuses relatively slowly into fast conducting motor fibres at low concentrations [14].

Selective sensory blockade, by preserving motor innervation, allows the patient to move his or her fingers. This helps in performing physiotherapy and improving venous blood flow, both of which are crucial to recovery.

Infraclavicular approach to brachial plexus may also be employed and a subcutaneously tunneled catheter may be placed, especially if it is anticipated that the patient will have difficulty in retaining the catheter without displacement. Accurate placement may be confirmed using a nerve stimulator or ultrasound guidance. In our case, as even the tactile stimuli were excruciating, peripheral nerve stimulator was not used.

Ropivacaine is another agent that may be considered for low concentration brachial plexus blockade. The degree of motor blockade produced by ropivacaine is less than that of bupivacaine. So it is possible to produce a more selective sensory blockade with ropivacaine. Furthermore, if required, higher concentration of ropivacaine may be used with lesser risk of cardiotoxicity than with bupivacaine [15].

Addition of fentanyl potentiates local anaesthetic action via central opioid receptor mediated analgesia by peripheral uptake of fentanyl to systemic circulation. Fentanyl also acts directly on the peripheral neuronal cells as the dorsal roots contain opioid binding sites. In addition, because of the presence of bidirectional axonal transport of opioid binding protein, fentanyl penetrates the nerve membrane and acts at the dorsal horn [16]. Adjuvants such as ketamine, alpha 2 adrenergic agonists have also been used to potentiate the effects of local anaesthetics in brachial plexus blocks [17].

Although brachial plexus blockade has been used for this condition [18], to the best of our knowledge, the use of low-dose bupivacaine (concentration and volume) that has the added advantage of preserving the motor function of patients to perform active physiotherapy has not been described before.

## Conclusion

Intravenous administration of phenytoin should be undertaken with care, ensuring a rate less than 50 mg per minute [9], through a free-flowing infusion. The management of PGS is primarily conservative. A continuous blockade of the brachial plexus with a low concentration of bupivacaine and fentanyl helps to alleviate the vasospasm and the pain while preserving the motor function for the patient to perform active movements of the finger and hand.

## Consent

Written informed consent was obtained from the patient for publication of this case report and accompanying images. A copy of the written consent is available for review by the Editor-in-Chief of this journal.

## Competing interests

The authors declare that they have no competing interests.

## Authors' contributions

GS treated the patient, documented the progress and outcome and prepared the initial manuscript. VTC performed the placement of the cannula, and was a major contributor in editing and revising the manuscript. BPT was the primary physician under whom the patient was admitted, supervised the hand therapy and further management of the patient and reviewed the manuscript. All authors read and approved the final manuscript.
